# Intranasal Delivery of Ivermectin Nanosystems as an Antitumor Agent: Focusing on Glioma Suppression

**DOI:** 10.1021/acsbiomaterials.5c00642

**Published:** 2025-06-11

**Authors:** Maiara Callegaro Velho, Valeria Luiza Winck, Camila da Silveira Mariot, Juliete Nathali Scholl, Augusto Ferreira Weber, Rita de Kássia Souza, Fernanda Visioli, Fabrício Figueiró, Monique Deon, Diogo André Pilger, Ruy Carlos Ruver Beck

**Affiliations:** † Programa de Pós-Graduação em Ciências Farmacêuticas, 28124 Universidade Federal do Rio Grande do Sul (UFRGS), Porto Alegre, Rio Grande do Sul (RS) 90620-170, Brazil; ‡ Laboratório de Nanocarreadores e Impressão 3D em Tecnologia Farmacêutica (Nano3D), 117303 Faculdade de Farmácia − UFRGS, Porto Alegre, RS 90620-170, Brazil; § Laboratório de Análises Bioquímicas e Citológicas (LABC), Faculdade de Farmácia − UFRGS, Porto Alegre, RS 90620-170, Brazil; ∥ Laboratório de Imunobioquímica do Câncer (LIBC), Departamento de Bioquímica − UFRGS, Porto Alegre, RS 90620-170, Brazil; ⊥ Programa de Pós-Graduação em Ciências Biológicas: Bioquímica, Instituto de Ciências Básicas da Saúde − UFRGS, Porto Alegre, RS 90620-170, Brazil; ∇ Departamento de Patologia Bucal, Faculdade de Odontologia − UFRGS, Porto Alegre, RS 90620-170, Brazil; # Programa de Pós-Graduação em Biociências, Universidade Federal de Ciências da Saúde de Porto Alegre (UFCSPA), Porto Alegre, RS 90620-170, Brazil

**Keywords:** cancer, glioblastoma, ivermectin, nose-brain, nanocapsules, mesoporous silica

## Abstract

Glioblastoma presents significant challenges in neuro-oncology due to its aggressive nature, drug resistance, and restrictions imposed by the blood–brain barrier. Ivermectin (IVM), known for its antiparasitic properties, has been highlighted as a promising treatment for tumors and an alternative therapy for glioma, although it exhibits low oral bioavailability. Therefore, we investigated the *in vivo* effect of IVM encapsulation in organic and inorganic nanosystems, first screened *in vitro* against different tumor cells and subsequently evaluated *in vitro* and *in vivo* glioma models. We produced IVM-loaded poly­(ε-caprolactone) nanocapsules (IVM-NC) using the interfacial deposition method, and IVM-loaded nanostructured silica particles (IVM-MCM) by loading IVM into commercial MCM-41 silica using the incipient wetness method. IVM-NC had a nanometric size (190 nm), a unimodal size distribution (span <2), and a high encapsulation efficiency (100% at 1 mg/mL). IVM-MCM exhibited a well-organized hexagonal mesoporous structure and high drug loading (0.12 mg/mg). Nanoencapsulated IVM significantly reduced the viability of various cancer cell lines, particularly glioma cell lines, which led us to evaluate them in a preclinical glioma model. We implanted adult male Wistar rats with C6 cells. Intranasal delivery of IVM-NC (60 μg/rat/day for 10 days) resulted in a larger decrease in tumor size compared with the group treated with free IVM, along with histopathological improvements. Treatment with IVM-MCM did not decrease the tumor size. However, both treatments were well-tolerated, with no adverse effects on weight, biochemical, or hematological parameters, or lung histology. Furthermore, the effective equivalent dose of IVM (26 μg/kg) in the rat glioma model was lower than the approved human dose for parasitic infections. This study marks the first exploration of IVM delivery to the brain. In summary, nasal administration of nanoencapsulated IVM via nanocapsules presents a promising avenue for targeted therapy against glioblastoma, with potential implications for clinical translation.

## Graphical abstract

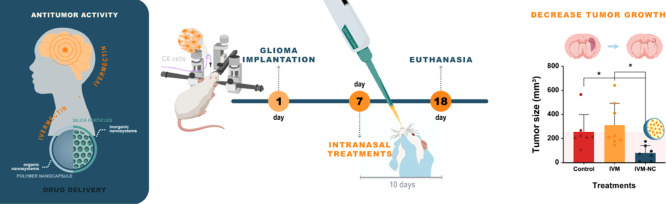

## 1 Introduction

The incidence of cancer is alarming: it is the second most common cause of death worldwide and is responsible for approximately 1 in 6 deaths.^1^ In 2022, an estimated 20 million new cancer cases and 9.7 million deaths occurred.^2^ This scenario is influenced by population aging, lifestyle habits, and environmental factors.^3^

Breast and lung cancers are the most commonly diagnosed around the world, while central nervous system (CNS) cancers rank 18th in incidence.^4^ Gliomas are the primary tumors of the CNS; they comprise approximately 60% of brain tumors.^5^ The classification of gliomas is based on the World Health Organization (WHO) system, with grades from I to IV. Lower-grade gliomas (grades II and III) exhibit an invasive nature and a high risk of progressing or recurring into high-grade gliomas (grade IV).^6^ Glioblastoma, grade IV, represents the most common and aggressive subtype, characterized by high invasiveness and multiple mutations. The prognosis is grim at best, with patients having an expected lifespan of approximately 15 months.^7^ Its anatomical location poses challenges for conventional therapy due to the presence of the blood-brain barrier (BBB) and mechanisms of multidrug resistance (MDR).^8^

Ivermectin (IVM) is an important drug for controlling parasitic infections due to its low cost and broad spectrum of activity.^9^ In the past decade, IVM has gained attention due to its exciting potential for drug repositioning, especially in cancer treatment. There is evidence of its multitarget antiproliferative and antitumor effects both *in vitro* and *in vivo* in various human cancer types.^10^ In breast adenocarcinoma, IVM demonstrated the ability to inhibit cell proliferation by inducing apoptosis and autophagy.^11^ Similar mechanisms have been reported in ovarian cancer cell lines,^12^ prostate cancer,^13^ colon cancer and melanoma.^14^ Additionally, IVM led to cell death through the induction of mitochondrial dysfunction and oxidative damage in human leukemia cell lines^13,15^ and renal cancer.^16^ Specifically, in glioma cells, the antiproliferative mechanisms of IVM have been associated with the induction of apoptosis through a caspase-dependent pathway,^17^ inhibition of the cell cycle in the G_0_/G_1_ phase,^18^ and inhibition of RNA helicase, thus blocking invasion and cell proliferation.^19^ Thus, IVM’s multitarget mechanism of action and safety profile make it a promising candidate for preclinical investigation. Nonetheless, the undesirable properties of IVM, such as low water solubility and limited bioavailability,^20^ hinder its suitability as a candidate drug and restrict its clinical application. Consequently, accurate selection and optimization of the formulation and route of administration for the treatment target are necessary.

The development of drug-delivery systems offers a promising strategy to enhance IVM’s antitumor efficacy. Furthermore, intranasal delivery, particularly using nanocarriers, presents an alternative approach to deliver drugs to the brain, increasing drug absorption and brain bioavailability. By circumventing the BBB through olfactory and trigeminal innervation, intranasal administration facilitates direct and rapid drug transport to the brain, overcoming the limitations of conventional delivery routes.^21^

Nanoparticle-based systems have demonstrated effectiveness for brain delivery via the nose-to-brain route, specifically by enhancing drug targeting and minimizing systemic distribution.^21–25^ In this scenario, organic and inorganic nanomaterials have been reported as carriers for efficient drug delivery to the brain via intranasal administration,^26^ including polymeric and lipid-based carriers.^27^ They are currently at the forefront of novel neuropharmacological treatments, demonstrating chemical versatility in encapsulating drugs and modifying their pharmacokinetics and biodistribution. In parallel, inorganic nanoparticles, with diverse compositions (e.g., silica, graphene, silver, gold, and iron oxide) and shapes (rods, prisms, spheres, and porous structures), offer a beneficial option for creating advanced drug-delivery systems.^26^

In our previous study, we focused on enhancing the biopharmaceutical properties of IVM to improve its antitumor efficacy at clinically relevant concentrations. We investigated the encapsulation of IVM in different nanostructured systems, including inorganic (nanostructured silica particles) and organic (poly-ε-caprolactone nanocapsules). Poly­(ε-caprolactone) (PCL) nanocapsules demonstrated higher encapsulation efficiency, better stability, and controlled release of IVM. Conversely, silica particles offered a high drug-loading capacity and enhanced the dissolution rate of IVM.^28^ In the present study, we assessed the impact of these nanocarriers on the *in vitro* antiproliferative effects of IVM against tumor cell models and their *in vivo* efficacy, using a preclinical glioma model in rats. Our investigation included screening to assess the viability of different cancer cell lines *in vitro* and evaluation of *in vivo* glioma growth suppression after intranasal administration. This study represents the first investigation of nose-to-brain delivery of IVM-loaded nanosystems for glioblastoma treatment. Additionally, we monitored the safety of the formulations by assessing hematological parameters, metabolic markers, hepatic markers, and histopathology.

## 2 Materials and Methods

### 2.1 Chemicals

IVM (97.5% purity, molecular weight = 875.1 g/mol) was obtained from Hebei Veyong Animal Pharmaceutical (Hebei, China). Regarding the excipients of the formulation, PCL and sorbitan monooleate (Span 80) were purchased from Sigma-Aldrich (São Paulo, Brazil), and medium-chain triglycerides and polysorbate 80 (Tween 80) were obtained from Delaware (Porto Alegre, Brazil) and Henrifarma (São Paulo, Brazil), respectively. Commercial silica type MCM-41 was purchased from Sigma-Aldrich (São Paulo, Brazil). Dulbecco’s Modified Eagle’s Medium (DMEM), penicillin/streptomycin, amphotericin, and the 4,5-dimethyl thiazol-2-yl-2,5-diphenyl tetrazolium (MTT) reagent were purchased from Sigma-Aldrich (St. Louis, USA), and fetal bovine serum (FBS) was obtained from Gibco (Grand Island, NY, USA).

### 2.2 Preparation and Physicochemical Characterization of Ivermectin-Loaded Poly­(ε-caprolactone) Nanocapsules

PCL nanocapsules were produced by interfacial deposition of preformed polymer.^28,29^ For the preparation of ivermectin-loaded nanocapsules (IVM-NC), an organic phase (a total volume of 27 mL) was formulated, dissolving 0.01 g of IVM, 165 μL of medium-chain triglycerides, and 0.1 g of PCL, with magnetic stirring at 40 °C for 4 h. Subsequently, the organic phase was injected into an aqueous phase (54 mL) containing 0.078 g of polysorbate 80 with continuous stirring, maintained for a period of 10 min. Finally, acetone was removed, and the suspension was concentrated under reduced pressure (Rotavapor *R*-100, Buchi, Flawil, Switzerland), until reaching a final volume of 10 mL and a concentration of 1 mg/mL IVM. After preparation, the particle sizes and size distributions (Span) were determined by laser diffraction (Mastersizer, Malvern Instruments, Malvern, UK), with the median diameter calculated based on the volume-weighted mean diameter (D_[4,3]_). Additionally, the mean hydrodynamic diameter of the particles (z-average) and the polydispersity index (PDI) were assessed using dynamic light scattering (Zetasizer Nano ZS, Malvern Instruments) after diluting the samples 500-fold in filtered ultrapure water (0.45 μm). The zeta potential (ZP) was measured by using the electrophoretic mobility technique in the same instrument, using samples diluted 500-fold in filtered 10 mM NaCl (0.45 μm). Finally, the pH of the nanocapsule suspension was assessed using a previously calibrated potentiometer (DM-22, Digimed, São Paulo, Brazil) immersed directly in the formulation. The IVM content was quantified using high-performance liquid chromatography (HPLC) based on a previously validated method (Velho et al. 2024). The chromatographic system included a liquid chromatograph (Shimadzu, Kyoto, Japan) equipped with a Shim-pack ClC Shimadzu column (4.6 mm × 250 mm, end-capped). The sample was eluted with a methanol:water mixture (90:10) at a flow rate of 1 mL/min. Drug extraction from IVM-NC was performed using methanol (20 μg/mL) with magnetic stirring for 6 min followed by vortexing for 2 min. Drug was detected at a wavelength of 254 nm, with a sample injection volume of 50 μL. Linear calibration curves for IVM were established in the range of 0.5–20 μg/mL (*r* > 0.99). The encapsulation efficiency (%EE) was determined after separation of IVM-NC from the IVM free fraction by ultrafiltration–centrifugation (0.45 μm pore size membrane, Millipore, Burlington, USA).

### 2.3 Preparation and Physicochemical Characterization of Ivermectin-Loaded Nanostructured Silica Particles

IVM was incorporated in commercial MCM-41 (hexagonal) nanostructured silica by using the incipient wetness method^30^ and an ethanolic solution of 20 mg/mL IVM, as described in our previous study.^28^ Then, 700 μL of this solution was added to 100 mg of previously dried MCM-41, equivalent to a drug loading of 12%. The sample was dried at room temperature for 72 h until total evaporation of the solvent. The mesoporous silica nanoparticles (MSNs) loaded with IVM were named IVM-MCM. The drug content of the IVM-MCM formulation was determined by HPLC according to the chromatographic conditions described in Section 2.2. IVM-MCM was dried at 90 °C for 2 h to eliminate moisture. Subsequently, 8 mg of the sample was mixed with 50 mL of methanol, subjected to ultrasonic agitation for 2 h at room temperature, followed by magnetic stirring for 1 h (750 rpm). The resulting suspension was centrifuged, and the collected supernatant was filtered (0.45 μm) before being injected into the chromatographic system. Thermogravimetric analysis (TGA) was used as a complementary method to evaluate the drug loading of IVM-MCM and its thermal stability. The analysis was conducted using a thermogravimetric analyzer (model TGA-50, Shimadzu, Kyoto, Japan). The samples were heated from room temperature to 900 °C at a rate of 20 °C/min, under an argon flow of 50 mL/min.

### 2.4 Morphological Analyses

IVM-NC and IVM-MCM were characterized morphologically by using a transmission electron microscope (JEOL JEM-1011, Peabody, USA) at an acceleration voltage of 100 kV, employing carbon-coated copper grids. IVM-MCM was dispersed in isopropanol, subjected to ultrasonic agitation for 5 min, and then applied to the grid. IVM-NC suspension was diluted 1:10 (v/v) in filtered ultrapure water. Uranyl acetate (2%, w/v) was used as a negative contrast.^31^

### 2.5 In Vitro Antiproliferative Effect and Cytotoxicity Evaluation

The *in vitro* antitumor screening of the IVM nanostructured systems was conducted using a human breast adenocarcinoma cell line (MCF-7), two human cervical carcinoma cell lines (HeLa and SiHa), and a rat glioma cell line (C6). Additionally, two healthy cell lines – fibroblast-like kidney cells (Vero) and fetal lung fibroblast cells (MRC-5) – were evaluated as nontumor controls to assess the cytotoxicity of IVM in the tested concentration range. The tumor cell lines were purchased from American Type Culture Collection (Rockville, MD, USA).

#### 2.5.1 Cell Culture

The cell lines were cultured in DMEM supplemented with 10% (v/v) FBS, containing 1% penicillin/streptomycin and 0.1% amphotericin. All cell lines were maintained in polystyrene flasks at 37 °C in a humid atmosphere containing 5% CO_2._ After determining the number of viable cells by staining with 0.4% trypan blue and counting in a Neubauer chamber, the cells were seeded in 96-well plates (5,000 cells/well). Twenty-4 h later, they were treated and incubated for 48 or 72 h (37 °C and 5% CO_2_). The treatments were described in Section 2.5.2.

#### 2.5.2 Treatments

The treatments consisted of IVM-NC, IVM-MCM, and nonencapsulated IVM at concentrations of 0.5, 1, 5, 10, and 25 μM of the drug. Formulations without the drug were also evaluated, namely plain nanocapsules (NC) and MCM-41 (silica particles) at a particle quantity equivalent to the highest concentration of each formulation. IVM-NC was diluted directly in the treatment wells, while IVM-MCM was suspended in phosphate-buffered saline (PBS) to reach a concentration of 1 mg/mL of the drug and subsequently diluted in the wells. For the preparation of free IVM solution, a stock solution was prepared with 16.4 mg/mL (16.7 mM) of the drug dissolved in dimethyl sulfoxide (DMSO) and subsequently diluted in PBS to reach a working concentration of 0.5 mg/mL. The maximum DMSO concentration used to treat cells was 0.6%; it was also assessed as vehicle control. Negative control (CN) received only culture medium. For this experiment, all nanocapsule suspensions were prepared under aseptic conditions to guarantee their sterility for safe biological evaluation. Silica particles underwent ultraviolet (UV) exposure (20 min) before the administration of each treatment to prevent any microbiological contamination.

#### 2.5.3 Cell Viability Assay

Cell viability was evaluated with the MTT assay.^32^ At the end of the treatment period (48 or 72 h), the supernatant was removed, and the wells were washed with PBS. Then, 100 μL of the MTT solution (0.5 mg/mL) was added to each well, and the plates were incubated for 4 h (37 °C and 5% CO_2_) protected from light. The supernatant was removed from the wells and the cells were resuspended in DMSO for solubilization of the formazan crystals formed by the reduction of MTT salt by the viable cells. Finally, the absorbance was measured at 570 and 630 nm with a spectrophotometer (Spectramax M2e and the SoftMax Pro Software Interface, Molecular Devices, Sunnyvale, CA, USA). The cytotoxic effect was expressed as the percentage of cell viability relative to the control cells (100%).

### 2.6 In Vivo Experimental Design

#### 2.6.1 Animals

Male Wistar rats (300–400 g, 10 weeks old) were used for the *in vivo* experiment, which was approved by the Ethics Committee of the Universidade Federal do Rio Grande do Sul (UFRGS, Porto Alegre, Brazil; protocol number: 43058) and performed in agreement with the guidelines given in “Principles of Laboratory Animal Care”. The rats were housed under controlled environmental conditions (12-h photoperiod at 22 ± 2 °C), with food and water provided *ad libitum*. The rats were acclimatized for 2 weeks before beginning the experiment.

#### 2.6.2 Sample Size

In a pilot study to determine the appropriate drug dose, the sample size was calculated based on the relevant literature.^24^ The predicted sample size, denoted as *n*, was set at 7 rats per group, including the IVM, IVM-NC, and IVM-MCM treatment groups. For tumor size, hematological, biochemical, and histopathological analysis, in accordance with previous studies involving a glioblastoma implant model in rats,^33^ the calculated sample size was adjusted to 10 rats per group. This resulted in a total of 81 rats for the complete experiment.

#### 2.6.3 Antitumor Treatments

In a pilot study, intranasal administration was validated for its ability to deliver the treatment to the brain and the appropriate drug dose. Rats (*n* = 7) were treated with IVM-NC, IVM-MCM, or an IVM solution, all at 1 mg/mL of drug. The rats were placed in the supine position, and 30 μL of the treatment was gently administered into each nostril using a micropipette, equivalent to 60 μg of drug per rat. After 1 h, the rats were euthanized for drug concentration analysis in the brain. For antitumor treatment, the rats were randomly divided into six treatment groups (*n* = 8–10 rats per group):

1. Control group – 5% DMSO in saline;

2. IVM group – IVM solution (1 mg/mL);

3. IVM-NC group – IVM-loaded nanocapsules (1 mg/mL of drug);

4. NC group – unloaded nanocapsules;

5. IVM-MCM group – IVM-loaded mesoporous silica particles (1 mg/mL of drug);

6. MCM-41 group – pristine silica particles.

Seven days postglioma implantation, a 10-day intranasal administration protocol was conducted according to a previously established method, delivering an IVM dose of 60 μg/rat in a total volume of 60 μL each day. The dose was determined based on the maximum recommended intranasal administration volume in rats and the tolerance observed during the pilot study, ensuring that no sneezing or rapid inhalation would occur. Additionally, the drug dose was restricted by the maximum IVM encapsulation in polymer nanocapsules, namely 0.1% (w/v, 1 mg/mL). On day 18 of the study (7 days postglioma implantation +10 days of treatment), the rats were euthanized using an isoflurane overdose. The brains were extracted for volume quantification and histopathological evaluation, the lungs were removed for histological assessment, and blood samples were collected for biochemical and hematological analyses.

### 2.7 In Vivo Glioma Model

Glioma implantation was conducted according to an established protocol.^33,34^ Briefly, C6 rat glioma cells (at approximately 80% confluence) underwent trypsinization, a single wash with DMEM, centrifugation, and suspension in the same medium. Male Wistar rats (300–400 g) were anesthetized via intraperitoneal (i.p.) administration of ketamine/xylazine (90 and 6 mg/kg, respectively). Subsequently, utilizing a Hamilton microsyringe coupled with an infusion pump (1 μL/min), approximately 3 × 10^5^ cells in a volume of 3 μL were injected at a depth of 6 mm into the right striatum (bregma coordinates: 0.5 mm posterior and 3 mm lateral). The rats were treated 7 days after implantation.

### 2.8 Tumor Volume and Histopathological Analysis

The extracted brains were fixed in 10% formalin and subsequently sectioned in the coronal plane into four slices from the point of tumor implantation (two slices forward and two slices reverse), each measuring 3 mm. Then, the slices were embedded in paraffin and again sectioned with a sliding microtome (3 μm thick) for hematoxylin and eosin (HandE) staining and subsequent histopathological analysis. Finally, images were captured using a digital camera connected to a microscope (BX-51, Olympus, Tokyo, Japan). The tumor area (mm^2^) was determined using the ImageJ software (National Institutes of Health, Bethesda, MD, USA). Then, the total tumor volume (mm^3^) was calculated by multiplying the area by the section thickness and adding up the volumes for the segmented areas.

A calibrated pathologist (kappa >0.75) who was blind to the treatment groups examined the HandE-stained brain sections. Assessment was based on parameters including intratumoral hemorrhage, necrosis, peritumoral edema, peripheral pseudopalisading, vascular proliferation, and lymphocytic infiltration, which were classified as absent or present. In each histological section, the segment with the highest tumor fraction was selected to analyze these parameters.

### 2.9 Preliminary Toxicity Evaluation

The body weight of each rat was measured periodically throughout the study to evaluate whether the treatment led to abnormal weight gain or loss. The weights were recorded on the day of tumor implantation surgery (day 0), before initiating treatment (day 7), and at the conclusion of treatment (day 18). To confirm that the treatments and the chosen route of administration did not induce pulmonary toxicity, lung tissues were removed, fixed in 10% formalin, routinely processed, and embedded in paraffin. Subsequently, 3 μm sections were obtained and stained with HandE. A pathologist blind to the treatment group conducted histological analysis to assess the presence or absence of hemorrhage, edema, inflammation, and necrosis. Blood samples were obtained by intracardiac puncture from the euthanized rats, collected in a tube with ethylenediaminetetraacetic acid (EDTA) and a clot activator tube. The objective was to assess hematological parameters and metabolic markers, including serum glucose and cholesterol. Additionally, hepatic markers, namely alanine aminotransferase (ALT) and aspartate aminotransferase (AST), and markers of kidney function, creatinine and urea, were evaluated.

### 2.10 Statistical Analysis

GraphPad Prism (GraphPad Software, La Jolla, CA, USA) was used for statistical analysis. Data are presented separately for each experimental protocol, expressed as mean ± standard deviation where applicable. Statistical significance was assessed using one-way analysis of variance (ANOVA). The *in vitro* assay data were subjected to analysis using the Dunnett and Tukey post hoc tests. The tumor volume data were preprocessed using the ROUT method with a Q coefficient of 1% to identify and remove outliers. Additional statistical analysis involved the nonparametric Kruskal–Wallis test. A p-value <0.05 was considered statistically significant.

## 3 Results

### 3.1 Ivermectin-Loaded Nanocapsules

IVM-NC formulations (*n* = 3) were prepared with a drug content of 1.02 ± 0.04 mg/mL (0.1% w/v), a high encapsulation efficiency of IVM (100%), and homogeneous macroscopic characteristics. The nanocapsules exhibited a unimodal granulometric distribution exclusively in the nanometric range, with a mean diameter (D_[4,3]_) of 187 ± 8 nm and a Span of 1.5 ± 0.1. According to the dynamic light scattering analyses, the IVM-NC displayed a Z-average of 213 ± 10 nm and a low PDI (0.13 ± 0.02). The nanocapsules also exhibited a negative ZP of – 11 ± 1 mV. The aqueous suspensions had a slightly acidic pH of 4.8 ± 0.01. The unloaded nanocapsules exhibited similar nanometric characteristics. Furthermore, transmission electron microscopy (Figure 1A) revealed spherical particles with regular, well-defined edges and an estimated average diameter of 400 nm.

**Figure 1:**
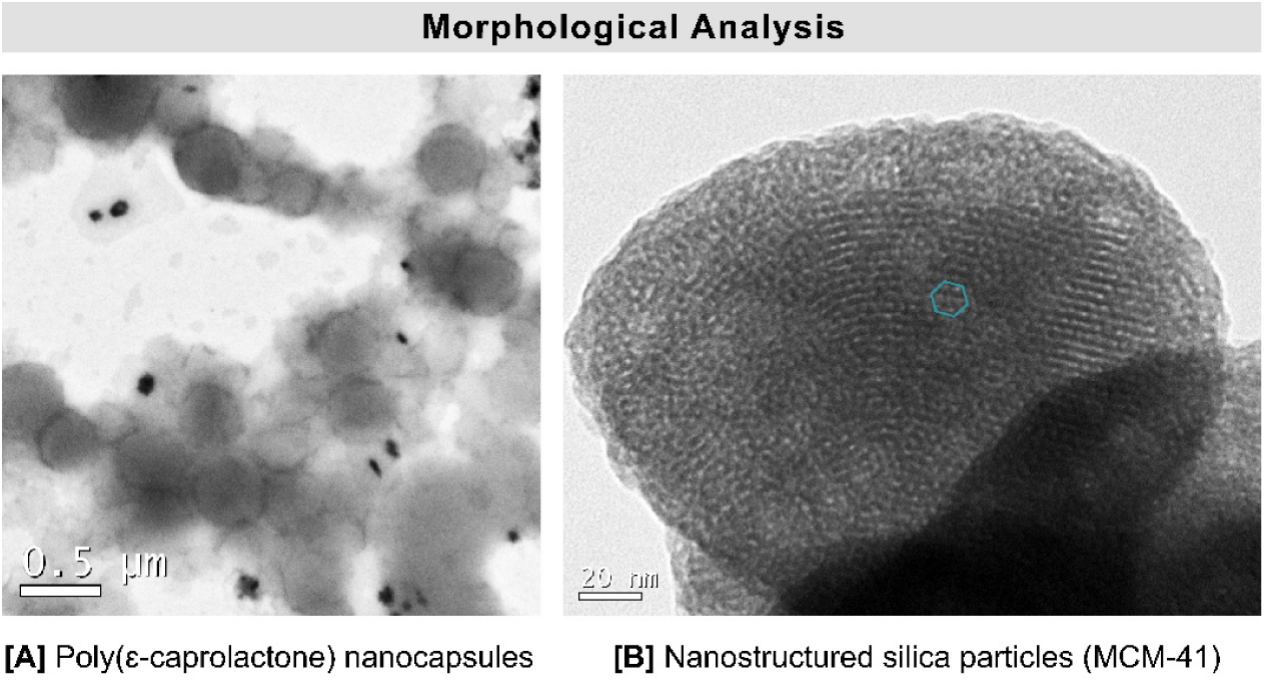
Morphological analysis by transmission electron microscopy. [A] Ivermectin-loaded nanocapsules (IVM-NC) with an image magnified 200,000×, and [B] mesoporous nanostructured silica (MCM-41) with an image magnified 300,000×. The green shape indicates the hexagonal arrangement of the pores.

### 3.2 Ivermectin-Loaded Nanostructured Silica Particles

Based on transmission electron microscopy analyses (Figure 1B), MCM-41 exhibited a hexagonal arrangement of pores in the nanometric range with regular organization. IVM-MCM had an estimated drug content of 0.12 mg/mg, representing a theoretical drug loading of 12% (w/w). The IVM content was 0.080 ± 0.004 mg/mg.

We used TGA as an additional method to assess drug loading in IVM-MCM. The thermal profiles of MCM-41, IVM-MCM, and IVM are shown in Figure 2. The initial mass loss up to 150 °C observed in all samples can be attributed to water desorption. The organic decomposition of IVM begins close to 300 °C, with total decomposition occurring above 450 °C.^35^ The silica samples (MCM-41 and IVM-MCM) exhibited a decline in mass above 600 °C due to the dehydroxylation process.^36^ The IVM-MCM thermogram showed a decrease in mass around 400 °C, suggesting that the loss of mass is related to the decomposition of IVM. We calculated the drug loading by subtracting the weight loss values of MCM-41 from IVM-MCM in the range of 150–800 °C. The mass loss was 11.5%, equivalent to a drug content of 0.116 mg/mg, validating the theoretical amount of IVM initially added to the formulation.

**Figure 2:**
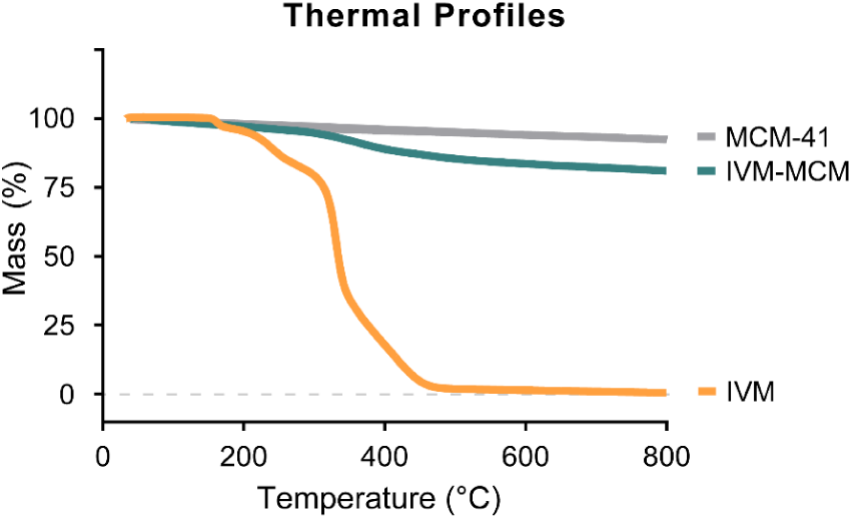
Thermogravimetric analysis of nanostructured silica (MCM-41), ivermectin-loaded silica particles (IVM-MCM), and pure ivermectin (IVM).

### 3.3 Cancer Cell Growth Inhibitory Effect

We assessed the viability of breast adenocarcinoma cells (MCF-7), cervical carcinoma cells (HeLa and SiHa), and glioma cells (C6) exposed to IVM-NC and IVM-MCM for 48 and 72 h by using the MTT assay. Treatment with IVM-NC had a less pronounced effect in inhibiting cell viability at 48 h (Figure 3). At the highest tested dose (25 μM), it demonstrated moderate cytotoxicity (viability of 69%–80%) for all tested tumor cell lines. Extending the exposure time to 72 h increased the inhibitory effect for HeLa and SiHa cells by 58% and 48%, respectively. Nevertheless, this represented a moderate degree of cytotoxicity.^37^ In contrast, for MCF-7 and C6 cells, the exposure time did not appear to influence the cytotoxic effect. Still, the half-maximal inhibitory concentration (IC_50_) decreased as the exposure time increased for HeLa cells (IC_50_ ∼ 25 μM) and SiHa cells (IC_50_ ∼ 22 μM).

**Figure 3:**
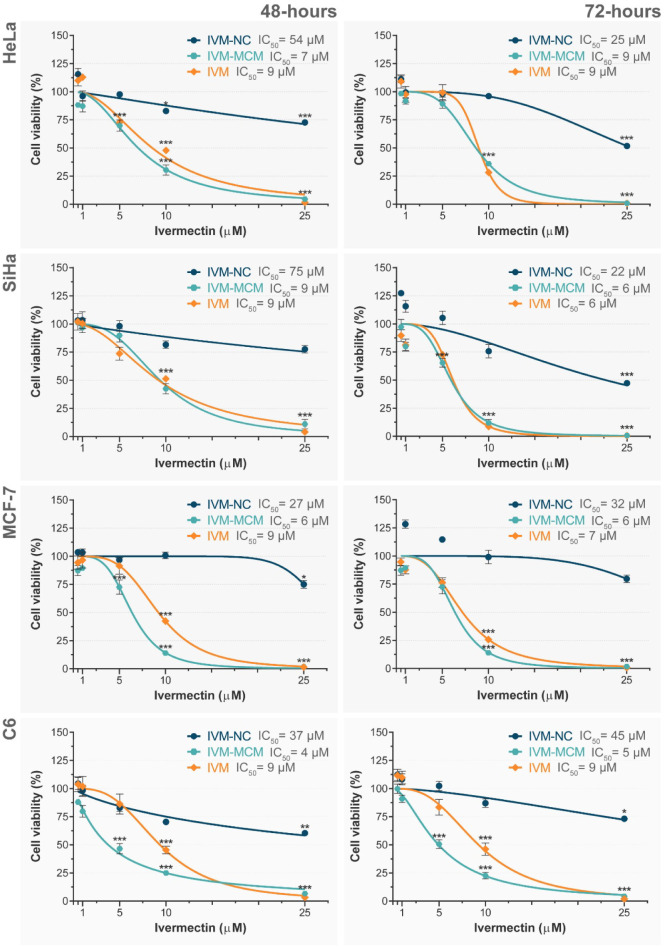
Viability of breast adenocarcinoma cells (MCF-7), cervical carcinoma cells (HeLa and SiHa), and glioma cells (C6) following exposure to nonencapsulated ivermectin (IVM), ivermectin-loaded nanocapsules (IVM-NC) and ivermectin-loaded silica particles (IVM-MCM) for 48 and 72 h. Data points represent treatment concentrations, and the corresponding IC_50_ curves are shown as lines. Results are expressed as the mean ± standard deviation (*n* = 4), relative to the negative control (100%). Asterisks (***) indicate statistically significant differences compared to the negative control, as determined by one-way ANOVA followed by Dunnett’s post hoc test (*p* < 0.001).

On the other hand, exposure to nonencapsulated IVM and IVM-MCM for 48 and 72 h led to a similar decline in viability across all tested tumor cell lines (Figure 3). Notably, there was moderate cytotoxicity (viability around 80%) at a concentration of 5 μM, showing a concentration-dependent cytotoxic response. There was complete inhibition at the highest tested dose (25 μM). In glioma cells, IVM-MCM (IC_50_ ∼ 5 μM) had a more pronounced cytotoxic effect compared to the nonencapsulated IVM (IC_50_ ∼ 9 μM).

We also investigated the effect of the nanoparticle *per se* on cancer cell death. We treated the cells with NC or MCM-41 in a particle volume equivalent to treatment with their homologous IVM-loaded particles with 25 μM of the drug. The response was consistent across all cell lines: NC slightly decreased cell viability, maintaining viability levels between 75% and 100%. In particular, in MCF-7 and HeLa cells, NC showed a similar effect to IVM-NC after 72 h of treatment. On the other hand, MCM-41 exhibited a notable cytotoxic effect after 48 h, reducing cell viability to 30–40%. Remarkably, this effect was reversed after 72 h of treatment, with cell viability recovering to approximately 80%. In the C6 cell line, MCM-41 reduced cell viability by about 20% after 72 h (Figure 3).

### 3.4 In Vivo Experiment

#### 3.4.1 Effects of Ivermectin-Loaded Nanoparticles on Suppressing Glioma Growth

We removed the brains on day 18 of the experiment – 7 days after implanting the glioma cells, followed by 10 days of intranasal treatment (IVM dose of 60 μg/day) – and analyzed the size and histopathological characteristics of the implanted tumors using routine HandE staining. IVM-NC reduced the tumor volume by 70% (residual tumor volume = 79 ± 23 mm^3^) compared with the control groups (254 ± 55 and 277 ± 76 mm^3^ for untreated and NC, respectively) (Figure 4). Conversely, free IVM and IVM-MCM did not significantly decrease tumor size (311 ± 64 and 174 ± 34 mm^3^, respectively) compared with the control groups (254 ± 55 and 345 ± 36 mm^3^ for untreated and MCM-41, respectively).

**Figure 4:**
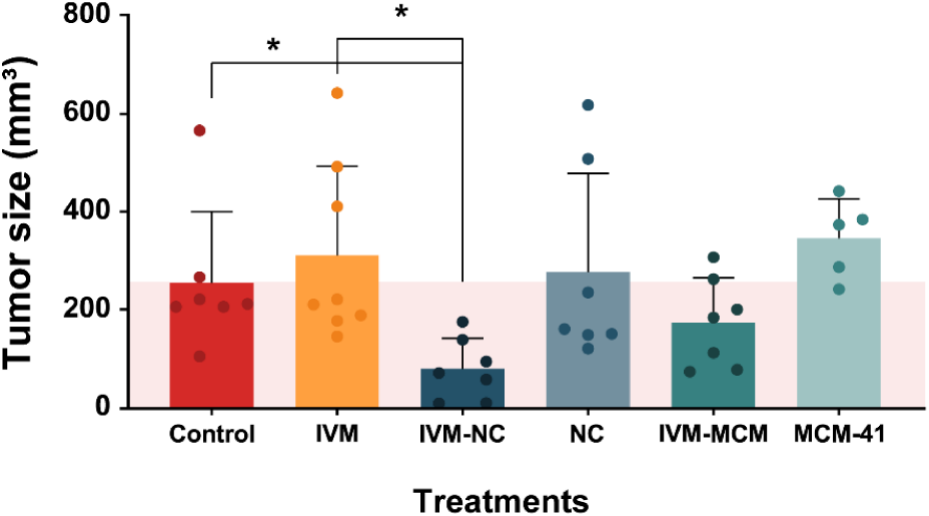
Quantification of the glioma tumor volume. Treatment groups: Control – saline; IVM – nonencapsulated ivermectin; IVM-NC – ivermectin-loaded nanocapsules; NC – nondrug-loaded nanocapsules; IVM-MCM – ivermectin-loaded silica particles; MCM-41 – nondrug-loaded silica particles. Variations in the sample size among the groups are attributable to the absence of tumor implantation in the rats from certain groups. The data were presented as the mean ± standard deviation and were analyzed using one-way analysis of variance followed by the Kruskal–Wallis test. * denotes a significant difference compared with the control and IVM groups (*p* < 0.05).

Histopathological analysis of the implanted tumors showed that necrosis, lymphocytic infiltration, intratumoral hemorrhage, peripheral palisading, and vascular proliferation were present in the control group (Table 1). While nonencapsulated IVM did not reduce the tumor volume, it reduced the incidence of necrosis, peritumoral edema, peripheral palisading, and vascular proliferation, indicating potential suppression of tumor invasion and aggressiveness.^38^ In addition to the reduction in the tumor volume, IVM-NC decreased the incidence of peritumoral edema and vascular proliferation. IVM-MCM treatment did not reverse the histopathological signs.

**Table 1:** Histological Characteristics of the Implanted Gliomas^a^

	Control	IVM	IVM-NC	NC	IVM-MCM	MCM-41
Intratumoral hemorrhage	6/8 (75%)	7/9 (78%)	6/8 (75%)	9/9 (100%)	7/9 (78%)	5/8 (62%)
Necrosis	6/8 (75%)	6/9 (67%) ↓	6/8 (75%)	7/9 (78%)	7/9 (78%)	5/8 (62%)
Peritumoral edema	4/8 (50%)	4/9 (44%) ↓	3/8 (37%) ↓	5/9 (55%)	6/9 (67%)	4/8 (50%)
Peripheric palisading	4/8 (50%)	3/9 (33%) ↓	5/8 (62%)	6/9 (67%)	6/9 (67%)	3/8 (37%)
Vascular proliferation	8/8 (100%)	8/9 (89%) ↓	7/8 (87%) ↓	8/9 (89%)	9/9 (100%)	8/8 (100%)
Lymphocytic infiltration	7/8 (87%)	9/9 (100%)	7/8 (87%)	9/9 (100%)	9/9 (100%)	8/8 (100%)

a Notes: The histological variables were expressed as percentages, considering hematoxylin and eosin–stained slides that present the histological variations relative to the sample number in the evaluated group. Treatment groups (*n* = 8–9): Control – saline; IVM – nonencapsulated ivermectin; IVM-NC – ivermectin-loaded nanocapsules; NC – nondrug-loaded nanocapsules; IVM-MCM – ivermectin-loaded silica particles; MCM-41 – nondrug-loaded silica particles. The down arrow (↓) indicates a decrease in the incidence of the histopathological parameter relative to the control group.

### 3.5 Preliminary Toxicity Evaluation

We assessed the safety of the IVM treatments *in vitro* and *in vivo*. Figure 5 shows the cytotoxic effects (based on the MTT assay) of both nonencapsulated and nanoencapsulated IVM in healthy cell lines, namely fibroblast-like kidney cells (Vero) and fetal lung fibroblast cells (MRC-5), treated for 72 h.

**Figure 5:**
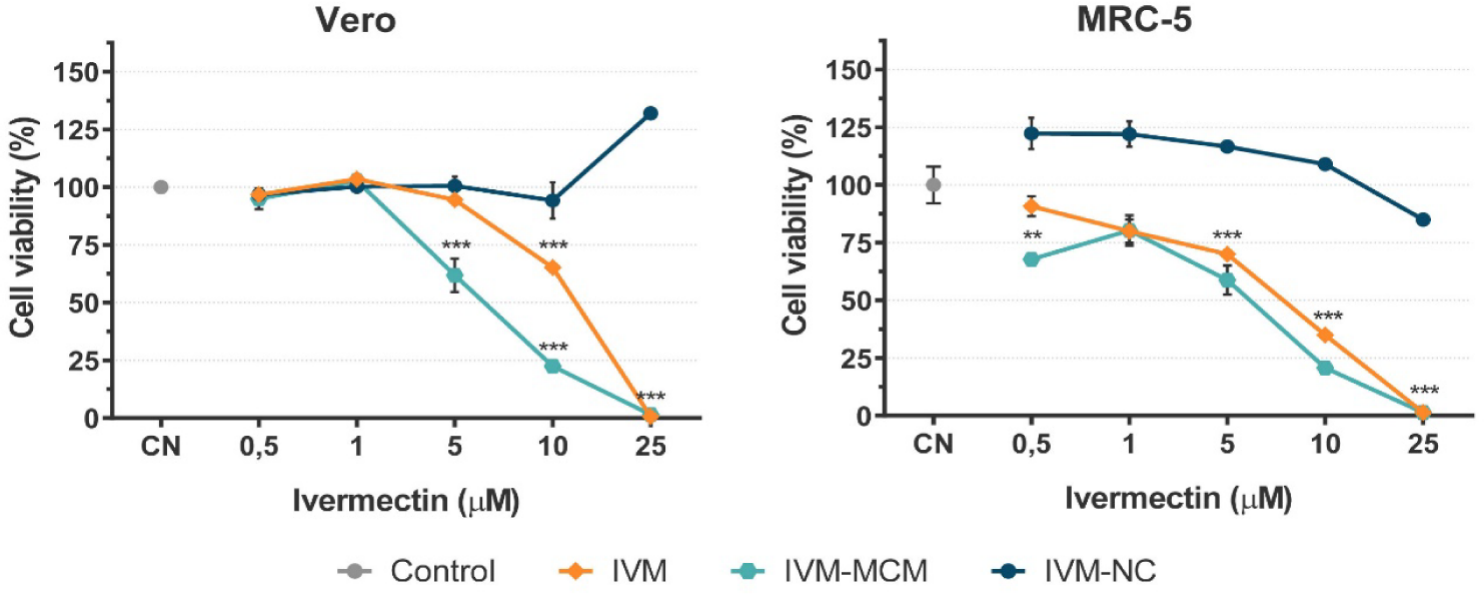
Evaluation of the cell viability of normal fibroblast lines (Vero and MRC-5) after incubation with nonencapsulated ivermectin (IVM), ivermectin-loaded nanocapsules (IVM-NC), and ivermectin-loaded silica particles (IVM-MCM) for 72 h. The results were expressed as the mean ± standard deviation (*n* = 4), percentage relative to the negative control (CN, 100%). Data points marked with *** indicate a significant difference based on the analysis of variance followed by Dunnett’s post hoc test (*p* < 0.001).

The tested IVM-NC concentrations were not cytotoxic. On the other hand, nonencapsulated IVM and IVM-MCM demonstrated significant cytotoxicity at higher concentrations in both cell lines. At the 10 μM drug concentration, IVM exhibited moderate cytotoxicity (65% cell viability) for Vero cells and high cytotoxicity (35% cell viability) for MRC-5 cells, while IVM-MCM showed high cytotoxicity in both cell lines (20% cell viability).^37^ There was complete inhibition of cell proliferation at 25 μM for both treatments.

During the *in vivo* experiment, we periodically measured each rat’s body weight from presurgery to tumor implantation and post-treatment. At the end of the 10-day treatment protocol, we removed the lungs for histological assessment and collected blood samples for biochemical and hematological analyses. The glioma-implanted rats did not exhibit significant differences in body weight among groups (Figure 6). The biochemical analysis, as presented in Table 2, did not indicate alterations in metabolic markers (glucose and cholesterol), hepatic markers (ALT and AST) or markers of kidney function (creatinine and urea) in the serum after 10 days of treatment. Furthermore, the hematological profile remained within the normal values observed for rat blood (Table 3).

**Figure 6:**
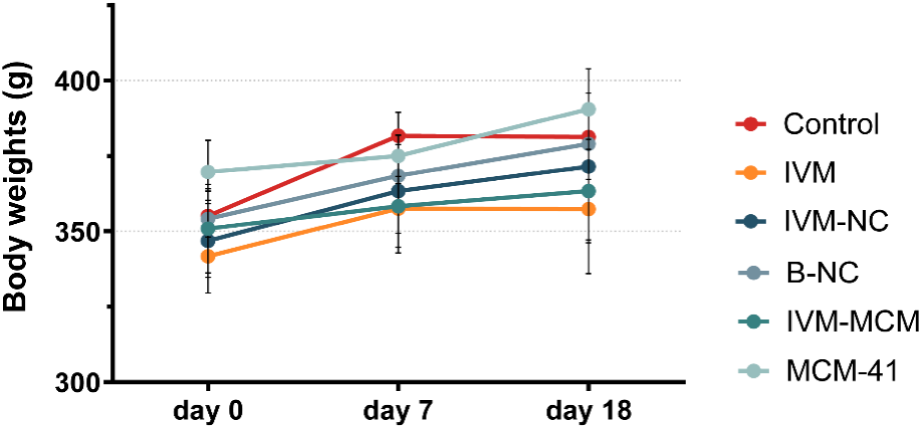
Body weight of Wistar rats over the 18-day experiment (presurgery, pretreatment, and post-treatment): saline (control), nonencapsulated ivermectin (IVM), ivermectin-loaded nanocapsules (IVM-NC), nondrug-loaded nanocapsules (NC), ivermectin-loaded silica particles (IVM-MCM), and nondrug-loaded silica particles (MCM-41). Data are presented as the mean ± standard deviation.

**Table 2:** Biochemical Parameters in Glioma-Implanted Rats Treated with Nonencapsulated and Nanoencapsulated Ivermectin^a^

	Control	IVM	IVM-NC	IVM-MCM
Glucose (mg/dL)	165 ± 17	183 ± 22	210 ± 33	246 ± 45
Cholesterol (mg/dL)	42 ± 5	46 ± 3	45 ± 7	46 ± 7
Alanine aminotransferase (U/L)	66 ± 7	56 ± 9	61 ± 9	62 ±4
Aspartate aminotransferase (U/L)	127 ± 8	141 ± 24	133 ± 31	126 ± 35
Creatinine (mg/dL)	0.56 ± 0.06	0.54 ± 0.05	0.56 ± 0.05	0.52 ± 0.06
Urea (mg/dL)	48 ± 4	48 ± 3	48 ± 3	49 ± 1

a Note: Data are presented as the mean ± standard deviation (*n* = 6). Treatment groups: Control – saline; IVM – nonencapsulated ivermectin; IVM-NC – ivermectin-loaded nanocapsules; IVM-MCM – ivermectin-loaded silica particles.

**Table 3:** Hematological Parameters in Glioma-Implanted Rats Treated with Nonencapsulated and Nanoencapsulated Ivermectin^a^

	Control	IVM	IVM-NC	IVM-MCM
White blood cells (×10^3^/μL)	2.7 ± 1.5	4.9 ± 0.7	4.4 ± 2.4	4.8 ± 1.8
Neutrophils (%)	10.4 ± 1.4	16.3 ± 1.7	14.9 ± 2.5	20.2 ± 2.7**
Lymphocytes (%)	88.2 ± 2.1	81.7 ±7.6	81.8 ± 2.6	76.6 ± 3*
Eosinophils (%)	0.1 ± 0.05	0.5 ±0.06	0.5 ±0.01	0.5 ± 0.01
Monocytes (%)	2.2 ± 1	1 ± 0.2	1.2 ± 0.4	2.8 ± 0.6
Red blood cells (×10^6^/μL)	10.1 ± 1.1	8.2 ± 0.7	8.6 ± 0.8	8.5 ± 0.3
Haematocrit (%)	61.1 ± 6.9	50.1 ± 3.2	51 ± 5.2	50.8 ± 2.2
RDW (%)	14 ± 0.6	13.7 ± 0.1	13.4 ± 0.5	13.8 ± 0.3
Platelets (×10^3^/μL)	657 ± 12	730 ± 70	657 ± 96	727 ± 28

a Note: Data are presented as the mean ± standard deviation (*n* = 3). Treatment groups: Control – saline; IVM – nonencapsulated ivermectin; IVM-NC – ivermectin-loaded nanocapsules; IVM-MCM – ivermectin-loaded silica particles. Data points marked with * or ** indicate a significant difference based on the analysis of variance followed by Dunnett’s post hoc test (*p* < 0.05 and *p* < 0.01, respectively).

On the other hand, histopathological examination of lung tissues revealed changes in the rats treated with nonencapsulated IVM, with foci of hemorrhage and inflammation (Table 4). The rats treated with IVM-NC did not show any histological changes, but the rats treated with IVM-MCM presented areas of hemorrhage and edema in the lung. Although inflammation was not evident in this group, neutrophilia based on the hematological analysis, compared with the control rats, could be attributed to the presence of edema.

**Table 4:** Histological Characteristics of the Lung in Glioma-Implanted Rats Treated with Nonencapsulated and Nanoencapsulated Ivermectin^a^

	Control	IVM	IVM-NC	NC	IVM-MCM	MCM-41
Hemorrhage	Absent	Absent	Absent	Absent	3/3 (100%)	Absent
Edema	Absent	2/3 (67%)	Absent	Absent	2/3 (67%)	3/3 (100%)
Inflammation	Absent	2/3 (67%)	Absent	Absent	Absent	Absent
Necrosis	Absent	Absent	Absent	Absent	Absent	Absent

a Notes: The histological variables were regarded as present or absent. Treatment groups: Control – saline; IVM – nonencapsulated ivermectin; IVM-NC – ivermectin-loaded nanocapsules; IVM-MCM – ivermectin-loaded silica particles.

## 4 Discussion

Previous studies have highlighted the clinical potential of IVM for treating glioma, a challenging neuro-oncological disease due to its aggressive nature and resistance to treatment.^39^ However, IVM’s efficacy is limited by its poor solubility and low oral bioavailability. To address this issue, we developed two different proposals to overcome these drawbacks and to enhance the IVM antitumor efficacy at clinically feasible concentrations.

Recently, in an extensive literature review, we discussed how micro/nanodrug-delivery systems can manipulate IVM’s physicochemical properties, providing evidence that, unequivocally, nanotechnology is an effective tool for improving the solubility and bioavailability of IVM, also covering the properties of targeting specific organs or tissues and facilitating sustained concentrations of the drug at the site of action.^9^ Experimentally, in a previous study, we investigated the encapsulation advantages and behavior of IVM in distinct nanostructured systems – organic (poly­(ε-caprolactone) nanocapsules) and inorganic (nanostructured silica particles) – to assess their influence on intrinsic solubility and modulation of the release profile *in vitro*. Our data indicated that nanoencapsulation of IVM in either organic or inorganic particles allows a marked improvement in the *in vitro* performance of the aqueous solubility of IVM. Furthermore, previous findings suggest that inorganic nanocarriers (IVM-MCM) offer a more efficient dissolution rate compared with organic carriers (IVM-NC).^28^

In the present study, we focused on understanding how the type of IVM nanocarrier and the drug release kinetics could impact the therapeutic antitumoral performance of IVM. Nanostructured carriers offer compelling advantages for the treatment of malignant solid tumors, including precise dosage control and enhanced therapeutic efficacy, achieved through pharmacokinetic modulation.^40^ Among the organic nanomaterials, polymeric nanocapsules stand out due to their biocompatibility, sustained drug release capacity, and improved permeability and retention at the tumor site.^41^ Conversely, inorganic nanocarriers, exemplified by MSNs, exhibit remarkable stability, a high drug-loading capacity, and easy functionalization.^25^ MSNs have gained prominence in pharmaceutical science, particularly as carriers for drugs with poor water solubility, that are used for cancer therapy.^42^

Here, we produced IVM-NC, which presented a homogeneous system without precipitate formation, and exhibited a uniform nanometric size distribution in the typical range for this type of nanoparticle (150–250 nm).^29,43^ Additionally, IVM-NC exhibited a negative ZP, which is attributed to the electronegative nature of the surface compounds, the presence of the PCL shell, and steric stabilization provided by polysorbate 80. The formulation also exhibited a slightly acidic pH, which is consistent with the properties of the polymer; anionic charges tend to acidify the formulation. Such features are commonly observed in PCL-based nanocapsules, whose pH typically ranges from 5 to 6.^44,45^ This pH range is well tolerated biologically, supporting the *in vivo* use of these formulations. The high encapsulation efficiency can be attributed to IVM’s high log P, allowing it to disperse well in the oily core of the nanocapsules while maintaining a drug concentration below the saturation point in the oil. The production of IVM-NC was consistent and reproducible, aligning with the parameters previously established for this nanoformulation.^28^ Efforts to develop novel IVM formulations using nanocarriers have been well documented, primarily focusing on treating parasitic diseases.^9^ Studies have shown that IVM-loaded polymeric nanoparticles exhibit promising properties, such as a particle size of 96–400 nm, a high encapsulation efficiency, and stable physicochemical characteristics.^46–48^

In parallel, we efficiently incorporated IVM into nanostructured silica particles, achieving a drug loading of 12% (w/w), as reported previously.^28^ MCM-41, with its hexagonal pore arrangement and high surface area, facilitated enhanced drug loading. Our earlier study indicated that IVM-MCM exhibited a reduced surface area and volume, consistent with pore-loading effects, with IVM stabilized in its amorphous form within the nanosized pores.^28^ Incomplete drug recovery (70%, w/w) through HPLC was likely due to strong adsorption between IVM and the silica pores.^49^ TGA confirmed that there was no substantial drug loss during the loading process (>96% drug recovery). IVM-MCM showed excellent physicochemical stability, maintaining its drug load for over 12 months, as confirmed by TGA. Notably, our research group is a pioneer in the use of nanostructured silica particles for IVM delivery.

Although there is growing interest in repurposing IVM for cancer treatment and a clear potential for nanocarriers to enhance its biopharmaceutical properties, the combination of these two approaches remains underexplored. Our findings contribute to filling this gap by demonstrating that optimizing IVM delivery through nanoencapsulation can improve its therapeutic efficacy, as supported by our cytotoxicity screening with several cancer cell lines and subsequent validation in an *in vivo* tumor model.

Treatment with the inorganic nanocarrier (IVM-MCM) decreased the viability of the breast adenocarcinoma (MCF-7), cervical carcinoma (HeLa and SiHa), and glioma (C6) cell lines in a concentration-dependent manner. At 25 μM, there was growth suppression. These results are comparable to the cytotoxic effect of nonencapsulated IVM. However, the IC_50_ indicated that half the concentration of IVM encapsulated in MCM (4–5 μM) is required to achieve the same antitumor efficacy as nonencapsulated IVM (8–9 μM). Conversely, treatment with the organic nanocarrier (IVM-NC) demonstrated moderate cytotoxicity for all tumor cell lines at the highest tested dose (25 μM). The divergent cytotoxic effects of nanostructured systems containing IVM can be attributed to variations in their drug release and diffusion rates, as recently reported by our group.^28^ IVM-MCM exhibited a higher drug dissolution rate (78% within 24 h) due to diffusion-based release through its pores. In contrast, IVM-NC demonstrated a more controlled release (30% within 24 h), requiring approximately 72 h for total drug release due to the permeability of the polymeric layer.^28^ As a result, effective inhibitory concentrations may not be reached within the first 48–72 h due to low free drug concentrations in the cellular medium.

Our *in vitro* findings align with previous reports indicating that IVM induces cell death in different cancer cell lines with a comparable IC_50_ values: 6–8 μM for HeLa cells,^50^ 1–5 μM for the U87 and T98G glioma cell lines,^51^ and 9–16 μM for C6 cells.^18^ Additionally, IVM exhibits cytotoxicity in MCF-7 cells.^11^ Particularly in glioma cell lines (U87, T98G, and C6), IVM exhibits a significant antiproliferative effect, inducing apoptosis via a caspase-dependent pathway, denoted by increased caspase-3 and caspase-9 activity,^18,51^ and by arresting the cell cycle in the G_0_/G_1_ phase.^18^ Furthermore, IVM induces mitochondrial dysfunction and oxidative stress, as reflected by the reduced mitochondrial respiration, membrane potential, and adenosine triphosphate (ATP) levels along with elevated mitochondrial superoxide production.^51,52^

Assessing the effects of nanomaterials without drugs is recommended to elucidate the intrinsic activity associated with the nanostructure.^43^ Thus, we evaluated the cytotoxicity of unloaded nanoparticles – pristine silica particles (MCM-41) and unloaded PCL nanocapsules (NC) – to distinguish the benefits of IVM nanoencapsulation. NC decreased cell viability, although to a lesser extent than IVM-NC. Specifically, in MCF-7 and HeLa cells, NC showed a similar effect to IVM-NC after 72 h. The intrinsic effects of the nanoparticles align with the premise that the drug release from IVM-NC failed to adequately inhibit these specific cells, indicating that the impact of IVM is less pronounced than that of the nanoparticles. MCM-41 exhibited marked cytotoxicity at 48 h, which was partially reversed at 72 h, with a shift to moderate toxicity levels. Notably, in the C6 cell line, cytotoxic effects remained severe even after 72 h. Although MCM-41 alone showed cytotoxic activity, its effect was less pronounced compared to IVM-MCM, highlighting the pharmacological activity of ivermectin. It has been hypothesized that a high concentration of nanoparticles may create a physical barrier over cells,^45^ hindering environmental exchange and causing accidental cell death, which is the result of a physical effect rather than a biological one.

Previous research supports our findings, showing that doxazosin-loaded NC exhibit enhanced antiproliferative effects against MCF-7 cells compared with free drugs.^53^ Similarly, tretinoin-loaded lipid-core nanocapsules demonstrated prolonged efficacy in HL60 cells,^54^ and encapsulating orlistat in nanocapsules amplified its cytotoxic effect against HeLa cells.^45^ Moreover, the potential of MSNs for poorly water-soluble drugs in cancer therapy is evident. For example, paclitaxel-loaded MSNs showed increased cytotoxicity against HepG2 liver carcinoma cells, achieving a significantly lower IC_50_ compared with paclitaxel solution.^55^ Additionally, codelivery of camptothecin and survivin using PEGylated MSNs enhanced cytotoxicity and induced apoptosis in C26 colorectal cancer cells.^56^ However, *in vitro* assays present limitations concerning the treatment times and drug concentrations; thus, the antitumor efficacy of IVM-NC cannot be dismissed compared with IVM-MCM and IVM treatments. This underscores the need for evaluation in more complex models. It is also essential to acknowledge that *in vitro* efficacy assessments in cancer cells are primarily focused on particle–cell interactions, often overlooking changes in biodistribution, mucoadhesion, and permeation through biological barriers. Therefore, based on the favorable *in vitro* results observed in the C6 cell line, including a lower IC_50_ and compelling evidence supporting IVM’s potential against glioma,^17^ −,^18,19,52^ we employed a preclinical glioma model using Wistar rats. Although prior *in vitro* and *in vivo* studies have investigated IVM’s application in glioma treatment, to the best of our knowledge, no study has specifically addressed the *in vivo* use of IVM-loaded nanosystems for targeted nose-to-brain delivery.

A significant challenge in glioblastoma treatment is achieving cerebral bioavailability of an effective chemotherapeutic drug. Overcoming the BBB is crucial; however, drug concentrations must be sustained and tolerable to inhibit tumor growth without causing damage to cerebral tissue. Intranasal administration exploits olfactory and trigeminal innervation and providing a potential route for the direct and rapid transport of drugs from the nasal cavity to the brain, bypassing the BBB.^57^ Consequently, intranasal administration is emerging as a promising noninvasive method for drug delivery to the brain. Drug-delivery nanosystems can enhance retention time in the nasal mucosa, facilitating drug absorption,^58^ and increasing bioavailability, particularly for poorly water-soluble,^24^ thus overcoming specific limitations inherent to this route.

Our treatment protocol involved administering low doses of IVM (164 μg/day) consecutively for 10 days to rats bearing glioma. The rats treated with IVM-NC exhibited a significant 3-fold reduction in tumor size compared to the control group (treated with 5% DMSO in saline). Additionally, there was a decreased incidence of key histopathological features of glioblastoma, including peritumoral edema and vascular proliferation. Notably, neither nonencapsulated IVM nor IVM-MCM significantly affected tumor size. Therefore, the observed tumor growth suppression can be attributed to the use of the organic nanocarrier as the delivery system.

A pharmacological response is directly influenced by the drug concentration at the target site. Our findings demonstrate a promising approach for nose-to-brain IVM transport using PCL-containing nanocapsules. This method effectively bypasses the selective brain barrier, facilitating successful drug delivery to the tumor site. We have shown how nanotechnology offers a promising strategy to improve the IVM delivery to the brain, while ensuring safety and efficacy in an *in vivo* glioma model.

The precise mechanisms underlying nanoparticle translocation across the BBB are not yet fully elucidated. Endothelial cell-mediated processes—such as surface protein recognition, endocytosis, modulation of tight junctions, and P-gp inhibition—have been strongly proposed.^59,60^ Moreover, key nanoparticle attributes—including size, polymer composition, and surface characteristics—play a pivotal role in steric stabilization, prolonged systemic circulation, and enhanced accumulation in brain tumors.^60,61^ Certain polymeric nanoparticles possess the ability to circumvent MDR mechanisms and enhance the retention of colloidal particles into the tumor interstitium.^38^ However, it is essential to acknowledge that, for many drugs, substantial absorption still occurs through the walls of nasal microvessels, in addition to the established olfactory and trigeminal absorption pathways.^62^ Moreover, the BBB consists of a monolayer of endothelial capillary cells with abundant P-glycoprotein (P-gp), an efflux transporter that contribute to the barrier by actively expelling drugs, such as IVM, from brain endothelial cells into the bloodstream.^26,63^

Consistent with our findings, previous studies have indicated that nasal delivery of simvastatin in PCL nanocapsules stabilized by polysorbate 80 enhanced drug permeability and transport to the brain compared with the drug solution.^23^ Similarly, sesame oil-based lipid carriers using polysorbate 80 improved central nervous system access, likely due to P-gp inhibition.^22^ Additional evidence supports these observations, as indomethacin encapsulated in PCL nanocapsules coated with polysorbate 80 achieved higher intracerebral concentrations than the drug solution.^64^ Notably, in glioma-implanted rats, indomethacin accumulation was more pronounced in the tumor-affected hemisphere than in the contralateral healthy side, suggesting that glioma-induced disruption of the BBB may facilitate drug penetration.^64^ The local disruption of the BBB, marked by the presence of capillaries and fenestrations, enables the enhanced permeability and retention (EPR) effect, facilitating the passive accumulation of nanostructured drugs at the tumor site.^64,65^

One of the most prominent aspects of our findings is the role of particle size in determining drug delivery efficiency to the brain via the nasal route. Previous studies have demonstrated that small-sized, drug-loaded nanoparticles, typically ranging from 100 to 400 nm, exhibit enhanced nose-to-brain transport.^21,60^ In our study, the differences in the *in vivo* efficacy observed between the IVM-loaded nanostructured systems may be attributed to their distinct size profiles: IVM-NC exhibited a narrow size distribution around 200 nm, whereas IVM-MCM displayed a broader distribution ranging from nanometers to micrometers. Supporting this, ponatinib-loaded MSNs with a narrow size range of approximately 100 nm showed an increase in the amount of drug delivery to the brain when administered intranasally compared with the free drug.^25^ Curcumin-loaded MSNs developed for nose-to-brain delivery exhibited uptake by olfactory cells at a nanoparticle size of <500 nm.^66^ Therefore, smaller particle sizes and larger surface areas enhance drug solubility, mucosal interaction, and drug permeation than drug solutions.^26^ This aspect should be considered in future studies to optimize the IVM-MCM particles evaluated here, either by controlling their size at the nanoscale or through functionalization.

Very few reports have addressed the *in vivo* antitumor efficacy of IVM in glioma models. The available studies have primarily utilized xenograft models with U251 cell implantation in BALB/c nude mice, outside the CNS. Mice treated with IVM (20 mg/kg, i.p.) exhibited slower tumor growth compared with those treated with saline, and elevated apoptosis markers, indicating IVM’s potential to suppress U251 cell growth and to induce autophagy at high doses.^17,18^ In our study, we evaluated a clinically relevant IVM dose that is below the typical approved a dose of approximately 200 μg/kg for use in humans. Each rat received a daily IVM dose of 164 μg/kg for 10 days, equivalent to 26 μg/kg in humans based on the Reagan-Shaw formula:^67^ Human Equivalent Dose = Animal Dose (mg/kg) × Rat Km/Human Km, where the Human and Rat Km are 37 and 6, respectively.

Our results also confirm that intranasal administration of IVM was safe and well-tolerated by the rats, as we did not observe significant changes in weight among the groups, and the biochemical parameters and hematological profiles remained within the normal values for rat peripheral blood. Our findings agree with a previous study^68^ and suggest that treatment with IVM at a dose of 60 μg/day was safe and well-tolerated by the rats independently of whether it was encapsulated and the drug-delivery system. Moreover, the preservation of normal lung histology implies the sustained normal functioning of the organ following treatment with IVM-NC. This is consistent with the cytotoxicity data obtained for the healthy cell lines, including MRC-5: IVM-NC was not cytotoxic at an IVM concentration of 25 μM.

It is important to note that the results obtained in the *in vitro* screening of tumor cells did not precisely reflect the *in vivo* antitumor effect. This discrepancy underscores the importance of a robust experimental design that includes complementary tests at distinct levels of complexity. The initially suggested antitumor potential for inorganic nanostructured particles (IVM-MCM) was surpassed by organic nanoparticles (IVM-NC) when evaluated in a model incorporating multiple intrinsic factors and extrapolating beyond a controlled microenvironment. Our preclinical investigation underscores the ability of intranasal IVM nanosystems, particularly polymeric nanocapsules, to facilitate minimally invasive nose-to-brain administration with effective drug delivery. Such a promising strategy aligns not only with the specific biopharmaceutical demands of IVM but also meets the clinical requirements for safety and efficacy in the treatment of glioma.

## 5 Conclusion

We designed innovative IVM formulations based on nanotechnological delivery systems for applications in oncotherapy, particularly for the treatment of glioblastoma. Specifically, polymeric nanocapsules allowed the efficient delivery of IVM to the brain, addressing the biopharmaceutical challenges of IVM, validating the intranasal route as a minimally invasive strategy and demonstrating a significant *in vivo* antitumor effect. We demonstrated the antitumor efficacy at doses lower than those used clinically for humans. Additionally, the treatments demonstrated good safety and tolerability. The potential of inorganic nanoparticles cannot be disregarded, but additional studies must be conducted to optimize their nanometer size range, to promote efficient permeation across the BBB and to achieve better drug bioavailability in the brain. In conclusion, nose-to-brain therapy targeted by IVM nanoencapsulation has shown promise against glioblastoma and the potential for clinical translation.
